# Emerging Molecular Technologies in Genitourinary Tumors

**DOI:** 10.3389/fonc.2018.00489

**Published:** 2018-10-30

**Authors:** Francesca Giunchi, Alessia Cimadamore, Michelangelo Fiorentino

**Affiliations:** ^1^Laboratory of Oncologic Molecular Pathology, S. Orsola-Malpighi Teaching Hospital University of Bologna, Bologna, Italy; ^2^Section of Pathological Anatomy, Polytechnic University of the Marche Region, School of Medicine, United Hospitals, Ancona, Italy

**Keywords:** next generation sequencing, targeted gene sequencing, NanoString System, patient-derived xenografts, organoids, renal cell carcinoma, prostate carcinoma, bladder carcinoma

## Introduction

Diagnostic molecular pathology of genito-urinary (GU) tumors is facing new technological challenges in the era of genome-wide analyses and patient-derived animal tumor models. In view of the increasing number of dedicated clinical trials, GU tumors represent the next urgent field of application of molecular diagnostics and drug discovery after gastro-intestinal and thoracic oncology.

## DNA-based genome-wide analyses

Wide spectrum mutational analyses using next generation sequencing (NGS) platforms will soon represent the standard-of-care technologies for the assessment of genetic variants in solid tumors (1). These technologies apply successfully to archival pathology specimens, cytological samples and even liquid biopsies (blood or pleural effusions) (2). Mutational analyses can be wider (whole exome sequencing, WES) or restricted to selected genes or amplicons (targeted gene sequencing TGS). Both approaches are used to identify single or multiple genetic variants as predictive biomarkers of response to targeted oncologic therapies. At least the following three genome-wide mutational analyses will become routine diagnostic tests for GU tumors in the immediate future. Analysis of *BRCA1* and *BRCA2* germ-line mutations will be required to assess inherited prostate cancer risk and to predict response to treatment with poly(ADP-ribose) polymerase (PARP) inhibitors and even next-generation anti-androgens (3, 4). Given the complexity of the *BRCA1* and *BRCA2* mutations the NGS sequencing is the ideal method for their assessment. Similarly, deep sequencing of the DNA mismatch repair genes will be required in patients with familiar prostate and colorectal cancer for suspected Lynch syndrome (3). Mutations in homologous recombination repair genes (ATM/BRCA1/2 specifically) is enriched in men with advanced clinical stage (≥ cT3) and higher Gleason grade groups (≥ 3) (5). Patients with metastatic castration-resistant prostate cancer whose tumors harbor homologous recombination DNA repair gene alterations, experience a different response to PARP inhibitor therapy. In particular, patients with cancer harboring DNA repair alterations in genes other than BRCA2 are often non-responders (6). The assessment of tumor mutation burden defined as the number of mutations per mega-base of tumor cell DNA is becoming the most relevant candidate biological predictor of response to immunotherapies targeting the PD-1/PD-L1 axis (7). Tumor mutation load can be achieved either by WES or by TGS using NGS dedicated panels covering at least 2 mega-bases of tumor DNA. Assessment of tumor mutation load is also prognostically relevant in metastatic renal cell cancer and in muscle-invasive bladder cancer (8–10). Finally, epigenetic changes, including CpG island hypermethylation can be investigated using genome-wide methylation NGS panels in the attempt to better stratify high-grade and low-grade disease (11).

## RNA-based genome-wide analyses

Genome-wide trascriptome analyses include gene expression profiling, miRNA and non-coding RNA profiling and RNA sequencing. In particular, RNA sequencing with high-throughput NGS platforms starting from RNA libraries allows simultaneous analysis of differential gene expression, allele-specific expression, splicing variants, and gene rearrangements (12). These analyses can also be done on RNA and DNA contained in small extracellular vesicles (EVs) that could be found in blood, urine, and other biological fluids (13). RNA abundance and sequence can be also investigated by array hybridization using platforms such as the NanoString System (14). Immediate clinical application of RNA sequencing to GU tumor include primarily the following fields of interest. The study of tumor immune micro-environment through the expression analysis of immune response genes is becoming important to assess tumor response to immune check-point inhibitors and BCG in bladder cancer (15, 16). The new molecular classification of muscle-invasive bladder cancer is largely based on gene expression profiling (17). Recognition of the molecular subtypes has prognostic and therapeutic implications for patients with advanced urothelial cancer. The assessment in the tumor tissue of the AR-V7 splicing variant of the androgen receptor (AR) gene is a predictor of poor response to anti-androgens and good response to chemo-therapy in castration-resistant prostate cancer (CRPC). The presence of AR splicing variants can be successfully investigated by RNA sequencing in prostate cancer tissue samples (18).

## Patient-derived animal models

Patient-derived xenografts (PDX) are mouse models where disaggregated cells or little fragments of human tumors are implanted into immunodeficient mice. The establishment of a PDX allows treating and monitoring the response to treatment of the original tumor *in vivo* in the mouse, instead of the patient, providing the best therapeutic selection at the same time (19). This procedure is ethically and commercially valuable since it spares pointless drug toxicity to the patient while saving money for oncological treatments that would be ineffective. Successful PDX establishment for monitoring response to treatment has been described in GU tumors (20). In CRPC there are available examples of PDX for treatment with abiraterone and enzalutamide as well as for a number of drugs in pre-clinical phase of development (21). In papillary type kidney cancer harboring *MET* mutations, there is evidence of successful treatment of PDX with Cabozantinib and other MET inhibitors (22, 23). PDX created using human bladder tumor tissues have been utilized to assess response rates to cisplatin or PI3K inhibitors (24). The success of PDX establishment is highly variable and depends on several tumor-related or animal-related factors. For instance, in a meta-analysis on bladder cancer, the tumor engraftment rate varied between 20 and 100% (24). In addition, several flaws can affect the reliability of PDX as surrogate models of original patients' tumors. Tumor histological appearance may change in the PDX frequently toward squamous or sarcomatoid or neuroendocrine differentiation. Cancer cell proliferative rates in PDX may increase as well as cancer mutations may turn out enriched or underestimated (25). On the other hand, host mice for PDX can be selected to be totally immunodeficient or “humanized” by forcing in the animals the expression of cytokines or injecting in the mouse bloodstream human bone marrow stem cells to re-create the tumor inflammatory microenvironment. Humanized PDX have been established for several tumor types but not yet for GU cancers (26).

Organoids are 3D cell-cultures recapitulating the natural complex environmental organization of a normal or a cancer tissue. They differ from the cell-lines that grow flat in 2D and lack the signal trafficking and the organization of a tissue (27). Organoids can be constructed from human cancer cells or tissues and can be utilized for testing the response to drugs (28). Compared to PDx, organoids are more amenable to grow but they are transient in nature and represent a methodological choice in-between cell-lines and animal xenografts. Organoid models have been created to trait rare phenotypes or genotypes of prostate cancer and to test their potential response to drugs, or to track evolution of bladder cancer (29, 30).

Patient-derived models are increasingly used to address questions in GU oncology. There are still limitations to the reliability of these models to actually guide patients' therapy. In addition, these model technologies require dedicated infrastructures (such as bio-banks, laboratories, and animal facilities) and experienced professionals. There are also several ethical restrictions to the use of model systems in different countries. Notwithstanding, PDX and organoids represent a fascinating opportunity to enhance cancer drug discovery and to provide more therapeutic options to cancer patients.

## Author contributions

MF: Conception and design; FG: Drafting the manuscript and review of the literature; AC: Critical revision of the manuscript.

### Conflict of interest statement

The authors declare that the research was conducted in the absence of any commercial or financial relationships that could be construed as a potential conflict of interest.
